# Spirulina Supplementation with High-Intensity Interval Training Decreases Adipokines Levels and Cardiovascular Risk Factors in Men with Obesity

**DOI:** 10.3390/nu15234891

**Published:** 2023-11-23

**Authors:** Rashmi Supriya, Maryam Delfan, Ayoub Saeidi, Seyedeh Somayeh Samaie, Maisa Hamed Al Kiyumi, Kurt A. Escobar, Ismail Laher, Katie M. Heinrich, Katja Weiss, Beat Knechtle, Hassane Zouhal

**Affiliations:** 1 Center For Health & Exercise Science Research, Department of Sport, Physical Education and Health, Faculty of Social Sciences, Hong Kong Baptist University, Kowloon Tong, Hong Kong SAR 999077, China; rashmisupriya@hkbu.edu.hk; 2 Department of Exercise Physiology, Faculty of Sport Sciences, Alzahra University, Tehran 15847-15414, Iran; 3 Department of Physical Education and Sport Sciences, Faculty of Humanities and Social Sciences, University of Kurdistan, Sanandaj, Kurdistan 66177-15175, Iran; saeidi_as68@yahoo.com; 4 Department of Exercise Physiology, Faculty of Physical Education and Sport Sciences, Alborz Campus, University of Tehran, Tehran 15719-14911, Iran; s.samaie@gmail.com; 5 Department of Family Medicine and Public Health, Sultan Qaboos University, Muscat P.O. Box 35, Oman; drmaysa@squ.edu.om; 6 Department of Family Medicine and Public Health, Sultan Qaboos University Hospital, Muscat P.O. Box 35, Oman; 7 Department of Kinesiology, California State University, Long Beach, CA 90840, USA; kurt.escobar@csulb.edu; 8 Department of Anesthesiology, Pharmacology, and Therapeutics, Faculty of Medicine, University of British Columbia, Vancouver, BC V6T 1Z3, Canada; ismail.laher@ubc.ca; 9 Department of Kinesiology, Kansas State University, Manhattan, KS 66502, USA; kmhphd@ksu.edu; 10 Research Department, the Phoenix, Manhattan, KS 66502, USA; 11 Institute of Primary Care, University of Zurich, 8091 Zurich, Switzerland; katja@weiss.co.com (K.W.); beat.knechtle@hispeed.ch (B.K.); 12 Medbase St. Gallen Am Vadianplatz, 9000 St. Gallen, Switzerland; 13 M2S (Laboratoire Mouvement, Sport, Santé)—EA 1274, Université de Rennes, 35000 Rennes, France; 14 Institut International des Sciences du Sport (2I2S), 35850 Irodouer, France

**Keywords:** cardiometabolic health, adipocytokines, Spirulina supplementation, metabolic complications

## Abstract

Adiposity, a state characterized by excessive accumulation of body fat, is closely linked to metabolic complications and the secretion of specific adipokines. This study explores the potential of exercise and Spirulina supplementation to mitigate these complications and modulate adipokine release associated with obesity. The primary objective of this investigation was to examine the impact of a 12-week regimen of high-intensity training combined with Spirulina supplementation on adipokine concentrations and lipid profiles in male individuals with obesity (N = 44). The participants were randomly distributed into four groups, each consisting of 11 participants: a control group (CG), a supplement group (SG), a training group (TG), and a training plus supplement group (TSG). The intervention comprised a 12-week treatment involving Spirulina supplementation (6 g capsule daily), a 12-week high-intensity interval training (HIIT) protocol with three sessions per week, or a combined approach. Following the interventions, metabolic parameters, anthropometric measurements, cardiorespiratory indices, and circulating adipokines [CRP, Sema3C, TNF-α, IL-6, MCP1, IL-8] were assessed within 48 h of the before and final training session. Statistical analyses revealed significant differences across all measures among the groups (*p* < 0.05). Notably, post hoc analyses indicated substantial disparities between the CG and the three interventional groups regarding body weight (*p* < 0.05). The combined training and supplementation approach led to noteworthy reductions in low-density lipoprotein (LDL), total cholesterol (TC), and triglyceride (TGL) levels (all *p* < 0.0001), coupled with an elevation in high-density lipoprotein–cholesterol (HDL-C) levels (*p* = 0.0001). Furthermore, adipokine levels significantly declined in the three intervention groups relative to the CG (*p* < 0.05). The findings from this 12-week study demonstrate that Spirulina supplementation in conjunction with high-intensity interval training reduced adipokine levels, improved body weight and BMI, and enhanced lipid profiles. This investigation underscores the potential of Spirulina supplementation and high-intensity interval training as a synergistic strategy to ameliorate obesity-related complications and enhance overall cardiometabolic well-being in obese males.

## 1. Introduction

Obesity exerts detrimental effects on a multitude of physiological systems by impairing the proper functioning of tissues and organs, thereby contributing to the onset of various diseases. It stands as a prominent risk factor for numerous noncommunicable disorders, including type 2 diabetes (T2D), cardiovascular ailments, hypertension, stroke, diverse forms of cancer, and mental health concerns [1]. Key factors in the genesis of obesity and its associated disorders, such as insulin resistance and T2D, encompass alterations in the levels of circulating adipokines and cytokines [2,3]. Adipokines represent bioactive molecules discharged into the bloodstream from adipose tissue, orchestrating metabolic changes in a manner that affects a wide array of tissues and organs [4]. Similarly, cytokines, predominantly released by white blood cells, wield systemic and localized influences [4]. In the context of obesity, inflammatory adipokines and cytokines often display heightened levels and have been implicated in the pathogenesis of various disease processes [1]. Prominent among the elevated obesity-associated circulating adipokines and cytokines are the C-reactive protein (CRP) [5,6], Semaphorin-3C (Sema3C), Tumor Necrosis Factor-alpha (TNF alpha), Interleukin-6 (IL-6) [7], Monocyte Chemoattractant Protein-1 (MCP1) [7], and Interleukin-8 (IL-8) [8].

Oxidative stress plays a pivotal role in mediating the escalation of adipokine-induced inflammation, with reactive oxygen species (ROS) increasing as visceral fat expands during the progression of obesity, thus modifying the expression and secretion of inflammatory adipokines [3,5]. ROS generation can stem from oxidative phosphorylation within the mitochondria, and the extent of production is contingent on mitochondrial function [6]. In physiological circumstances, antioxidant buffering balances ROS production. However, in situations of overnutrition and obesity, ROS production may surpass the capacity of antioxidant buffering, culminating in oxidative stress wherein ROS disrupts cellular and tissue function, including the activation of pathways that augment adipokine and cytokine expression [3,6]. Notably, ROS plays a crucial role in developing obesity-linked cardiometabolic disorders [6].

Consequently, a growing interest has been in mitigating ROS levels in obesity. Several investigations have illustrated that dietary antioxidants, counteracting the pro-oxidative state of cells, might hold therapeutic potential for addressing obesity and its concomitant comorbidities [9]. Exogenous dietary antioxidants synergize with endogenous counterparts to bolster cellular antioxidant capacity [10]. Spirulina, an ancient cyanobacterium and one of the earliest life forms on Earth, boasts antioxidant attributes. Evidence underscores Spirulina’s potential to reduce blood lipid levels, body fat, waist circumference, body mass index, and appetite [11,12,13]. Nonetheless, the impact of Spirulina on oxidative stress and inflammation markers in humans remains a subject of debate [14,15].

Furthermore, the interplay between Spirulina supplementation and exercise, known to alleviate oxidative stress and inflammation markers, remains unclear [16,17]. Although acute exercise initially elevates ROS, regular exercise activates the endogenous antioxidant system, affording protection against oxidative damage [17,18]. A reduction in ROS levels could potentially modulate the altered profiles of adipokines and cytokines witnessed in obesity [17]. Notably, Spirulina supplementation has been shown to enhance acute exercise performance, fat oxidation, and glutathione levels while attenuating the rise in lipid peroxidation prompted by aerobic exercise [19,20,21]. In a particular study, the combination of High-Intensity Interval Training (HIIT) and Spirulina supplementation positively impacted immunoglobulin levels, cardiorespiratory fitness, and body composition in overweight and obese women, along with an increase in immunoglobulin A (IgA), vital for the immune system [19]. Prior research has also examined Spirulina’s influence on nesfatin-1, omentin-1, and lipid profiles among obese and overweight females [22]. The combination of Spirulina and HIIT elevated nesfatin-1 and omentin-1 levels, although Spirulina supplementation alone did not [22]. Another study scrutinized the independent and synergistic effects of Spirulina supplementation (4.5 g per day), with or without engagement in aerobic exercise three days per week and HIIT two days per week, on blood lipids and body mass index (BMI) in 52 sedentary men with excess body weight over six weeks [23]. The findings demonstrated that Spirulina supplementation potentiated the hypolipidemic effects of an intensive physical training regimen in males with excess body weight and dyslipidemia [23]. In past studies, it has been shown that Spirulina alone causes weight loss [13,24], and on the other hand, the combination of Spirulina consumption with exercise training reduces inflammatory indicators in obese people [25].

However, despite these observations pointing toward favorable impacts of Spirulina and HIIT on cardiometabolic health markers among individuals with obesity, alterations in adipokines during such interventions have yet to be investigated.

Consequently, the present study aims to explore the effects of a 12-week regimen combining HIIT and Spirulina supplementation on markers of cardiometabolic health, anthropometric measures, cardiorespiratory fitness, as well as adipokines and cytokines, in comparison to the effects of HIIT and Spirulina interventions in isolation. It is postulated that the combined intervention will yield more pronounced improvements in the measured variables compared to each intervention alone.

## 2. Methods

### 2.1. Ethical Approval

The research received approval from the Ethics Committee of the Sport Sciences Research Institute (Ethics code: IR.SSRC.REC.1401.093). All protocols adhered to the most recent iteration of the Declaration of Helsinki [19].

### 2.2. Participants

Following outreach in public spaces, laboratories, sports clubs, and social networks, 143 adult men volunteered to partake in the current research. Among them, 80 participants were eligible for the study entry criteria. Inclusion criteria: having a BMI exceeding 30 kg/m², lacking involvement in regular physical activities over the past six months, having no history of cardiovascular or endocrine disorders, and no smoking and alcohol consumption. Prospective participants with joint ailments, physical disabilities, and those utilizing prescription medications or supplements with potential impacts on muscle and adipose tissue metabolism were excluded from the study.

A subset of 80 individuals was ultimately chosen to participate after thoroughly evaluating the volunteers. The inclusion criteria necessitated that all participants undergo a comprehensive physical examination conducted by a qualified medical professional and clinical exercise physiologist during the initial visit. After the initial evaluation and explanation of different parts of the research, 64 individuals were selected. The sample size was based on the standardized effect size (SES) calculated using the mean and standard deviation values of similar studies reported in the literature [26]. The standardized effect size was placed in the G*Power (3.1.9.4) analysis program [two-sided, α = 0.05, power (1-β) = 0.95, effect size = 1.43]. Accordingly, the minimum sample number per group was determined to be nine. In the present study, we considered 16 subjects for each group. Additionally, participants were required to furnish a signed consent form and complete the Physical Activity Readiness Questionnaire (PAR-Q) [11]. This process ensured adherence to established research standards and ethical guidelines.

### 2.3. Experimental Design

**Figure 1:**
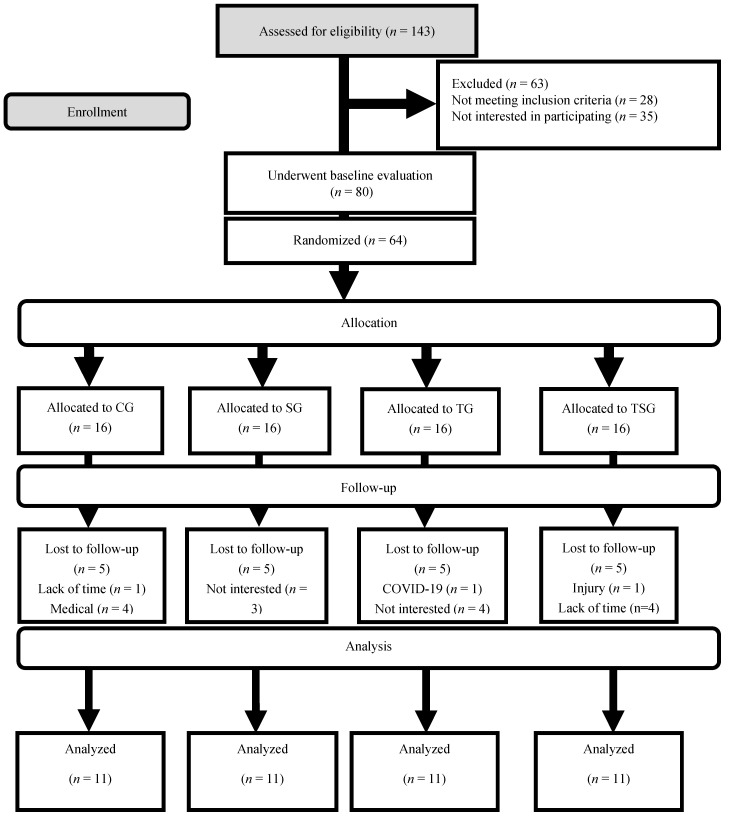
Flow of participant recruitment.

Participants underwent a familiarization session during which all study procedures were comprehensively elucidated, taking place one week before the initiation of the training regimens. Measurements of height and body mass were conducted for each participant. Subsequently, participants were assigned at random to one of four equitably sized groups (16 participants in each group): the Control Group (CG), the Supplement Group (SG), the Training Group (TG), and the Training + Supplement Group (TSG) Figure 1.

Throughout the study duration, 20 participants withdrew from the study due to medical conditions, work-related challenges, and a lack of sustained interest in the research. As a result, each group had 11 participants remaining for the subsequent analysis. Detailed instructions regarding the execution of training protocols were given to each group during the third session, coinciding with measurements of body mass and VO_2peak_.

Following the baseline measurements, the two groups engaged in training (TG and TSG) embarked on a 12-week exercise program involving three sessions per week. Participants allocated to all groups were instructed to maintain their lifestyles throughout the study. All measurements were conducted at consistent times of day (with a deviation of approximately 1 h) and under uniform environmental conditions (~20 °C and ~55% humidity).

Baseline assessments were conducted 48 h before the commencement of the training protocols, while post-tests occurred 48 h after the final session for all groups. Those participating in the training protocols were instructed to adhere to the same dietary regimen for 48 h before the baseline assessment and the concluding measurements.

### 2.4. Anthropometric and Cardio-Respiratory Fitness Assessments

The measurement of participants’ body mass and height was carried out using a calibrated scale (Seca, Halmburger, Germany) for weight and a stadiometer (Seca, Halmburger, Germany) for height. These measurements were subsequently utilized to calculate the participants’ body mass index (BMI) in kilograms per square meter (kg/m²). The assessment of VO_2peak_ was conducted employing a modified Bruce protocol within a controlled environment set at a temperature range of 21–23 °C. This protocol has been previously documented in studies involving individuals with overweight and obesity [11,12]. The exercise was performed on a motorized treadmill (H/P/Cosmos, Pulsar med 3p-Sports and Medical, Nussdorf-Traunstein, Germany).

The determination of VO_2peak_ adhered to the physiological criteria outlined by the American College of Sports Medicine (ACSM) guidelines. These criteria included reaching a point of perceived physical exhaustion and maximal effort, as indicated by participants’ responses on the Borg scale, or the identification of severe dyspnea, dizziness, and other constraining symptoms by the supervisor, following the ACSM and American Heart Association (AHA) guidelines for cardiopulmonary exercise testing (CPET) [27,28]. A plateau in both VO_2_ and respiratory exchange ratio (RER) ≥1.10 was used to confirm VO_2peak_ attainment.

Blood pressure readings were obtained using an electronic sphygmomanometer (Kenz BPM AM 300P CE, Nagoya, Japan), and heart rate was continuously monitored throughout the tests using a Polar V800 heart monitor (Finland). Gas analysis was carried out using a gas analyzer system (Metalyzer 3B analyzer, Cortex: biophysics, GmbH, Leipzig, Germany), which underwent calibration before each testing session.

### 2.5. Training Protocols

VO_2peak_ values were used to prescribe exercise intensity in training sessions, which consisted of treadmill running for 32 min. Prior to each training session, subjects performed 5 min of warm-up activities (stretching movements, walking, and running). In the first week, subjects ran on a treadmill at 65% of VO_2peak_ and in the second week at 75% of VO_2peak_ for 32 min and three sessions a week. In week three, the HIIT sessions began. In the third and fourth weeks, subjects performed intervals of 4 min of running at 75% VO_2peak_ followed by 4 min of inactive recovery for 32 min. In weeks 5, 6, and 7, subjects performed intervals of 4 min at 85% of VO_2peak_ with 4 min active recovery intervals at 15% of VO_2peak_ 32 min. In weeks 8, 9, and 10, subjects performed 4 min intervals at 90% VO_2peak_ with active recovery intervals at 30% VO_2peak_ for 4 min on the treadmill for 32 min. In weeks 11 and 12, subjects completed 4 min intervals at 95% VO_2peak_ with 4 min active rest intervals at 50% VO_2peak_ for 32 min. After completing the training in each session, subjects performed a 5 min cool-down at 50% VO_2peak_ in each session [29]. The control group continued their normal daily activities and were restricted from participating in regular physical activity.

### 2.6. Supplementation of Spirulina and Placebo

Spirulina samples (Hellenic Spirulina Net: Production unit: Thermopigi, Sidorokastro, Serres, Greece) were encapsulated for administration. Each subject ingested a total of 6 g daily, divided into two doses of 3 g each, one in the morning and the other in the evening, over 12 weeks [30]. Comparable amounts of placebo were also provided to both the Control Group (CG) and Treatment Group (TG). The placebo was formulated using corn starch, tinted with a food-grade green coloring resembling Spirulina powder, and enhanced with the essence of kiwi fruit for flavor. Corn starch, a neutral and inert substance lacking therapeutic properties, was selected for its established safety profile. Widely employed across the culinary and pharmaceutical sectors, it is recognized as a colorless, tasteless, secure, non-toxic, non-irritating, and hypoallergenic powder. Adherence to the supplementation regimen was defined as consumption of =80% of the assigned supplements by each participant.

### 2.7. Nutrient Intake and Dietary Analysis

Three-day food records (two weekdays and one weekend day) were obtained before and after the study to assess changes in habitual dietary intake over time [31]. Each food item was individually entered into Diet Analysis Plus version 10 (Cengage, Boston, MA, USA), and total energy consumption and the amount of energy derived from proteins, fats, and carbohydrates were determined (Table 1).

### 2.8. Blood Markers

All testing was carried out under standard conditions between 8:00 and 10:00 a.m. After 12 h of fasting, venous blood samples were taken from the right arm 48 h before the first exercise session and 48 h after the last session. Blood samples were transferred to EDTA-containing tubes, centrifuged for 10 min at 3000 rpm, and stored at −80 °C. Plasma total cholesterol (TC) and triglyceride (TGL) were measured using enzymatic methods (CHOD-PAP); high-density cholesterol (HDL-C) and low-density cholesterol (LDL-C) were determined using a photometric method (Pars Testee’s Quantitative Detection kit, Tehran, Iran) with a coefficient and sensitivity of 1.8% and 1 mg/dL and 1.2% and 1 mg/dL respectively. The hs-CRP levels were measured with an ELISA kit (Diagnostic Biochem, London, ON, Canada). Sensitivity: 10 ng/mL. Plasma TNF-α levels were measured with an ELISA kit (Elabscience Biotechnology, Wuhan, China). Catalogue No: E-EL-H0109. Sensitivity: 4.69 pg/mL. Intra-CV = 6.22%, inter-CV = 5.2%. Plasma IL-6 levels were measured with an ELISA kit (Biovendor, Brno, Czech Republic). Catalogue No: RD194015200R. Sensitivity: 0.65 pg/mL. Intra-CV = 4.7%, inter-CV = 4.9%. Plasma Sema3C levels were measured with an ELISA kit (MyBioSource, San Diego, CA, USA). Catalogue No: MBS2883689. Sensitivity: 2.3~40 ng/mL. Plasma MCP-1 levels were measured with an ELISA kit (R&D Systems, USA). Catalogue No: DCP00. Sensitivity: 10 pg/mL. Intra-CV = 7.8%, inter-CV = 6.7%. Plasma IL-8 levels were measured with an ELISA kit (citeab, Bath, UK). Catalogue No: 900-K18. Sensitivity: <7.5%.

### 2.9. Statistical Analysis

Descriptive statistics (means ± standard deviation) were used to describe all data. The normality of the data was assessed using the Shapiro–Wilk test. A two-way ANOVA repeated measures test was used to determine the Group × time interaction. One-way ANOVA and Fisher LSD post hoc tests were used for the evaluation baseline data of four groups. When a significant difference was detected using ANOVA, mean differences were determined using pairwise comparisons. The sample size was calculated to detect a statistical difference between study variables with a 95% confidence interval (CI) equal to or greater than 80% of the power value. Additionally, effect sizes (ES) were reported as partial eta-squared. In accordance with Hopkins et al. (2009) [32], ES were considered trivial (<0.2), small (0.2–0.6), moderate (0.6–1.2), large (1.2–2.0), and very large (2.0–4.0). A *p*-value of <0.05 was used to indicate statistical significance. All data were evaluated with SPSS software (version 24).

## 3. Results

### 3.1. Anthropometry and VO_2peak_

The four groups’ baseline differences were insignificant for body mass (*p* = 0.46) and BMI (*p* = 0.46). Body mass was significantly different from baseline in the TSG (*p* = 0.039); however, it was not in the SG (*p* = 0.72), TG (*p* = 0.12), or CG group (*p* = 0.70) (Table 1). BMI was not significantly different from baseline for any of the four groups (*p* > 0.05) (Table 2). There were no significant interactions between group and time for either weight (*p* = 0.28, η^2^ = 0.08) or BMI (*p* = 0.36, η^2^ = 0.07).

There were no significant differences between groups in VO_2peak_ values at baseline (*p* = 0.10). There were significant increases from baseline in the TG (*p* = 0.001) and TSG (*p* = 0.0001) after 12 weeks, but no changes in the CG (*p* = 0.29) or SG (*p* = 0.15). The interaction between time and groups was significant for VO_2peak_ (*p* = 0.001, η^2^ = 0.35). In comparison to the CG, after 12 weeks, VO_2peak_ was significantly higher in the TG (*p* = 0.003) and TSG (*p* = 0.001) but not in the SG (*p* = 0.51). There were no differences in VO_2peak_ between the SG and TG (*p* = 0.30) or between the TSG and TG (*p* = 0.99) or SG (*p* = 0.15) (Table 2).

### 3.2. Lipid Profiles

There were no differences between groups at baseline for HDL (*p* = 0.55), LDL (*p* = 0.94), TC (*p* = 0.72), and TG (*p* = 0.92). Compared to the baseline, HDL was significantly increased in the SG (*p* = 0.0001), TG (*p* = 0.0001), and TSG (*p* = 0.0001) but not in the CG (*p* = 0.07). The time × group interaction was significant for HDL (*p* = 0.0001, η^2^ = 0.65). The results of the Bonferroni test showed significant differences in the TG (*p* = 0.044) and TSG (*p* = 0.0001) compared to the CG but no difference between the SG (*p* = 0.09) and CG. Also, the changes in HDL in the TSG were significantly higher compared to the SG (*p* = 0.0001) and TG (*p* = 0.0001), but the differences between the SG and TG were not significant (*p* = 0.99) (Table 2).

Post-test values for LDL were significantly lower in the SG (*p* = 0.005), TG (*p* = 0.0001), and TSG (*p* = 0.0001) but not in the CG (*p* = 0.82) compared to baseline. The interaction between time and groups was significant for LDL (*p* = 0.0001, η^2^ = 0.63). Post hoc tests revealed that the reduction in LDL was significantly greater in the TG (*p* = 0.0001) and TSG (*p* = 0.0001) compared to the CG; however, it was not different between the SG and CG (*p* = 0.34). The reductions in LDL in the TG (*p* = 0.032) and TSG (*p* = 0.0001) were significantly greater compared to the SG, while the TSG was not different compared to the TG (*p* = 0.066) (Table 2).

Compared to the baseline, there were decreases in TC in the SG (*p* = 0.009), TG (*p* = 0.001), and TSG (*p* = 0.0001) following the 12-week intervention. There were no significant changes in TC at 12 weeks in the CG (*p* = 0.19). There was a significant interaction between time and groups for TC (*p* = 0.0001, η^2^ = 0.36). Results of the Bonferroni test showed that after 12 weeks, compared to the CG, TC was significantly lower in the SG (*p* = 0.039), TG (*p* = 0.002), and TSG (*p* = 0.001). There were no significant differences in TC at 12 weeks between the SG, TG, and TSG groups (*p* = 0.99) (Table 2).

Compared to baseline, TGL at 12 weeks was significantly lower in the SG (*p* = 0.006), TG (*p* = 0.0001), and TSG (*p* = 0.0001). There was a significant interaction between time and groups for TGL (*p* = 0.0001, η^2^ = 0.46). Results of the Bonferroni test showed that after 12 weeks, TGL was significantly lower in the TG (*p* = 0.003) and TSG (*p* = 0.001) compared to the CG. However, the SG was not significantly lower compared to the CG (*p* = 0.99). The reductions in TGL after 12 weeks were more significant in the TSG compared to the SG (*p* = 0.001) but not compared to TG (*p* = 0.99) (Table 2).

### 3.3. Adipokines and Cytokines

**Figure 2:**
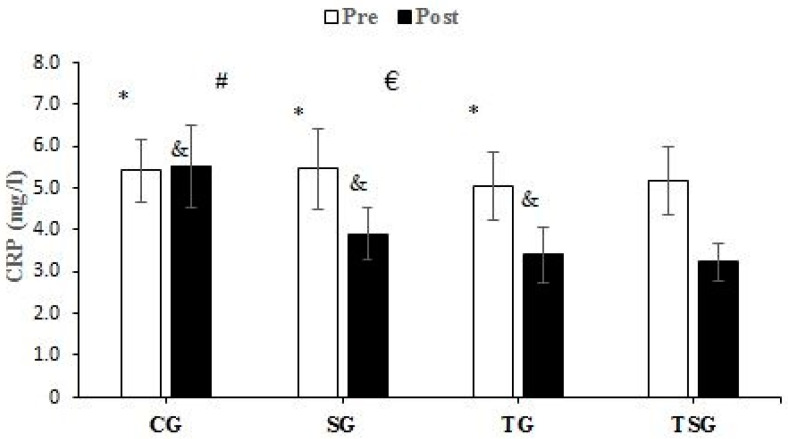
Pre- and post-training values (mean ± SD) for CRP in the Control (CG), Supplement (SG), Training (TG), and Training + Supplement (TSG) groups. & Significant differences with pretest values (*p* < 0.05). * Significant differences with the control group (*p* < 0.05). # Significant interaction between time and groups (*p* < 0.05). € Significant difference between TG and SG (*p* < 0.05).

**Figure 3:**
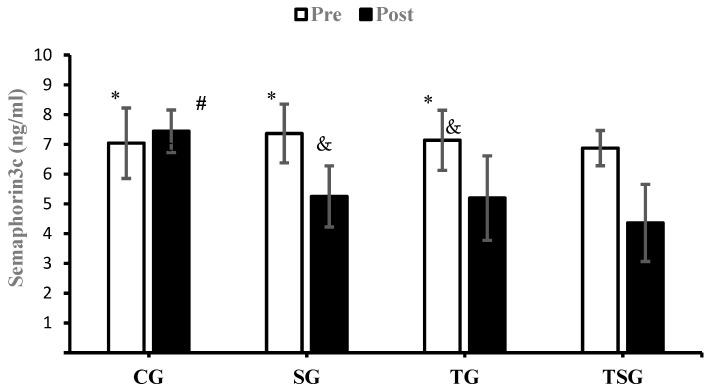
Pre- and post-training values (mean ± SD) for Semaphorin3c in control (CG), Supplement (SG), Training (TG), and Training + Supplement (TSG) groups. & Significant differences with pretest values (*p* < 0.05). * Significant differences with the control group (*p* < 0.05). # Significant interaction between time and groups (*p* < 0.05).

**Figure 4:**
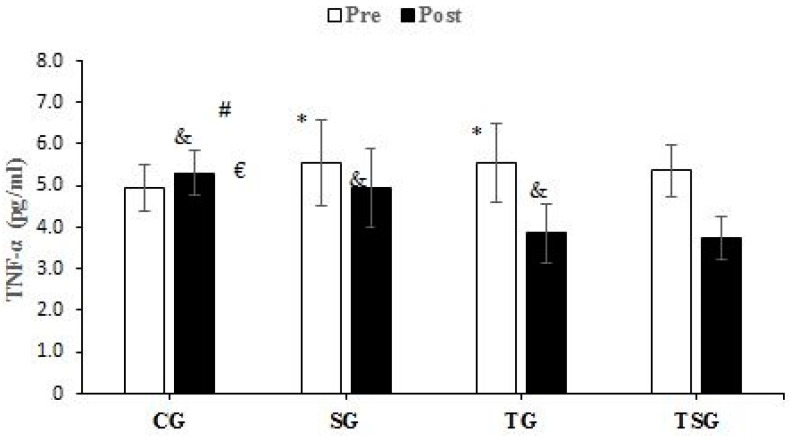
Pre- and post-training values (mean ± SD) for TNF-α in Control (CG), Supplement (SG), training (TG), and training + Supplement (TSG) groups. & Significant differences with pretest values (*p* < 0.05). * Significant differences with the control group (*p* < 0.05). # Significant interaction between time and groups (*p* < 0.05). € Significant difference between TG and SG (*p* < 0.05).

**Figure 5:**
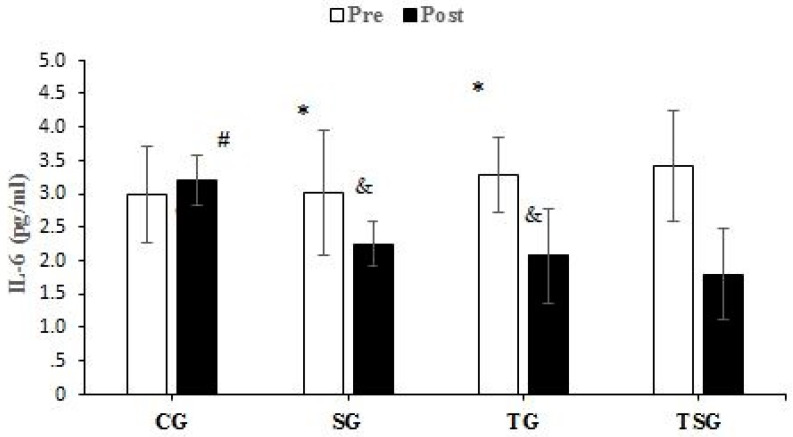
Pre- and post-training values (mean ± SD) for IL-6 in control (CG), Supplement (SG), training (TG), and Training + Supplement (TSG) groups. & Significant differences with pretest values (*p* < 0.05). * Significant differences with the control group (*p* < 0.05). # Significant interaction between time and groups (*p* < 0.05).

**Figure 6:**
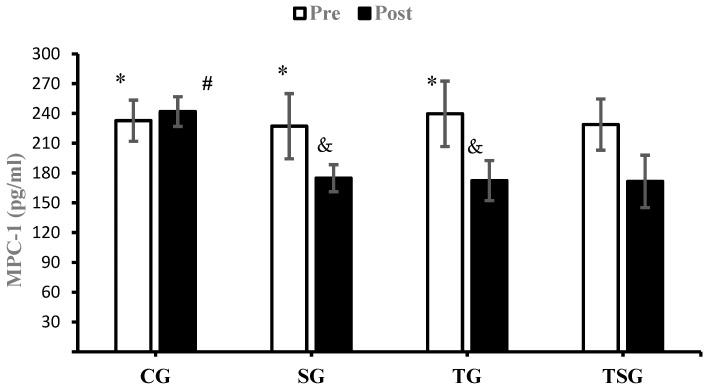
Pre- and post-training values (mean ± SD) for MCP-1 in control (CG), Supplement (SG), training (TG), and Training + Supplement (TSG) groups. & Significant differences with pretest values (*p* < 0.05). * Significant differences with the control group (*p* < 0.05). # Significant interaction between time and groups (*p* < 0.05).

**Figure 7:**
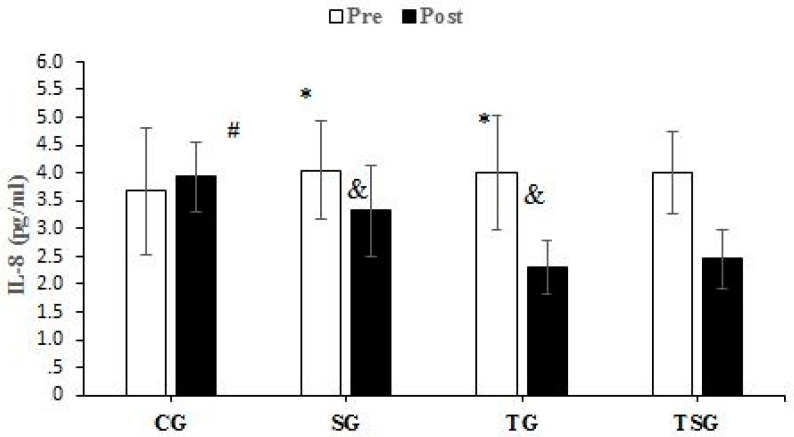
Pre- and post-training values (mean ± SD) for IL-8 in control (CG), Supplement (SG), training (TG), and Training + Supplement (TSG) groups. & Significant differences with pretest values (*p* < 0.05). * Significant differences with the control group (*p* < 0.05). # Significant interaction between time and groups (*p* < 0.05).

There were no significant differences between the groups in baseline values for CRP (*p* = 0.62), Sema3C (*p* = 0.68), TNF-α (*p* = 0.26), IL-6 (*p* = 0.50), MCP1 (*p* = 0.74), and IL-8 (*p* = 0.78). Compared to baseline, CRP was lower in the SG (*p* = 0.0001), TG (*p* = 0.0001), and TSG (*p* = 0.0001) but not in the CG (*p* = 0.72) after 12 weeks. There was a significant time × group interaction for CRP (*p* = 0.0001, η^2^ = 0.42). Post hoc test results showed that after 12 weeks, the CRP was lower in the SG (*p* = 0.002), TG (*p* = 0.001), and TSG (*p* = 0.0001) compared to the CG. CRP levels in the TSG were lower compared to the SG (*p* = 0.99) and TG (*p* = 0.99). CRP levels at 12 weeks were not different between the TG and SG (*p* = 0.99) (Figure 2). Sema3C at 12 weeks was significantly lower in the SG (*p* = 0.0001), TG (*p* = 0.0001), and TSG (*p* = 0.0001) compared to the baseline. There was no difference in the CG (*p* = 0.37) at 12 weeks compared to the baseline. Also, the time × groups interaction for Sema3C was significant (*p* = 0.0001, η^2^ = 0.40). The results of the Bonferroni test showed that after 12 weeks, compared to the CG, Sema3C was lower in the SG (*p* = 0.001), TG (*p* = 0.003), and TSG (*p* = 0.0001). However, there were no differences in Sema3C between the SG, TG, and TSG groups (*p* = 0.99) (Figure 3). Following the 12-week intervention, TNF-α was not different in the CG compared to the baseline (*p* = 0.17). However, TNF-α was lower in the SG (*p* = 0.0001), TG (T = 0.0001), and TSG (*p* = 0.0001) compared to the baseline. The interaction between time and groups was also significant for TNF-α (*p* = 0.0001, η^2^ = 0.52). The results of the Bonferroni revealed that at 12 weeks, TNF-α was significantly lower in the TG (*p* = 0.0001) and TSG (*p* = 0.0001) compared to the CG. There was no difference between the SG and CG (*p* = 0.057). Compared to the SG, TNF-α was significantly lower in the TG (*p* = 0.030); however, TNF-α levels in the TSG were not different from the SG (*p* = 0.057) and TG (*p* = 0.99) (Figure 4). Compared to the baseline, IL-6 was not different in the CG (*p* = 0.46) at 12 weeks, while concentrations were lower in the SG (*p* = 0.008), TG (*p* = 0.0001), and TSG (*p* = 0.0001). The time × groups interaction was also significant for IL-6 (*p* = 0.0001, η^2^ = 0.38). IL-6 was significantly lower in the TG (*p* = 0.004) and TSG (*p* = 0.0001) compared to the CG but not in the SG (*p* = 0.096). The reduction in IL-6 in the TSG was not different compared to the TG (*p* = 0.99) and SG (*p* = 0.19), while there was also no difference between the SG and TG (*p* = 0.99) (Figure 5). Compared to the baseline, MCP-1 at 12 weeks was significantly increased in the CG (*p* = 0.46), while MCP-1 was decreased in the SG (*p* = 0.0001), TG (*p* = 0.0001), and TSG (*p* = 0.0001). Also, a significant interaction between time and groups was observed for MCP-1 (*p* = 0.0001, η^2^ = 0.44). The results of the Bonferroni post hoc test showed that compared to the CG, MCP-1 was lower in the SG (*p* = 0.001), TG (*p* = 0.0001), and TSG (*p* = 0.001). There were no differences in MCP-1 between the SG, TG, and TSG groups (*p* = 0.99) (Figure 6). IL-8 concentrations at 12 weeks were significantly lower in the TG (*p* = 0.0001) and TSG (*p* = 0.0001) but were not different in the SG (*p* = 0.06) or CG (*p* = 0.50). Also, a significant interaction between time and groups was reported for IL-8 (*p* = 0.003, η^2^ = 0.30). The results of the Bonferroni test showed that IL-8 was significantly lower in the TG (*p* = 0.005) and TSG (*p* = 0.01) compared to the CG but not in the SG (*p* = 0.46). There were no significant differences at 12 weeks between the SG, TG, and TSG groups (*p* > 0.05) (Figure 7).

## 4. Discussion

This study demonstrated that 12 weeks of HIIT and Spirulina supplementation, separately and in combination, can improve circulating adipokines levels in obese men. The combination of HIIT and Spirulina supplementation overall led to greater changes in measured outcomes compared to each intervention alone. In this study, it was shown that HIIT and Spirulina supplementation decreased plasma levels of IL-8, IL-6, MCP-1, Semaphorin3c, CRP, LDL, TC, TGL, and TNF-α and increased HDL. We also showed that HIIT and Spirulina supplementation increases VO_2peak_ and decreases BMI.

Metabolic syndrome and other cardiovascular risk factors are strongly correlated with excess body fat [27]. As a result, global healthcare systems annually support new programs to reduce obesity and other cardiovascular hazards [28]. This study provides evidence for the beneficial effects of Spirulina supplementation (6 g/day) and 32 min of HIIT three times per week for 12 weeks on improving anthropometric measurements, cardiometabolic risk factors (TC, LDL, HDL, and TGL), and reducing pro-inflammatory adipokines (CRP, TNF-α, Sema-3C, IL-8, IL-6, and MCP-1) with the combination of HIIT and Spirulina improving cardiometabolic health makers greater than HIIT or Spirulina supplementation individually.

### 4.1. Favorable Modulations of Metabolic Factors and Cardiorespiratory Parameters

We found that 12 weeks of HIIT and Spirulina supplementation resulted in numerous improved markers of metabolic health. The most significant improvements came when training and supplementation were combined compared to the control group. Cardiorespiratory fitness is strongly related to cardiometabolic health and all-cause mortality [33]. Here, we show that HIIT increased VO_2peak_; however, the addition of Spirulina did not further increase VO_2peak_ compared to HIIT alone. The current literature on aerobic exercise performance and Spirulina supplementation is equivocal, with some evidence indicating it may possess an ergogenic effect while other evidence suggests it does not [34].

Similarly, blood lipid profiles were improved in all intervention groups; however, the most considerable improvement occurred in TSG vs. TG and SG. Reductions in TC, LDL, and TGL occurred with training and supplementation alone and to a greater degree when combined, while an increase in HDL was observed. While there are data demonstrating lipid-lowering effects of Spirulina supplementation [35] as well as a plethora of literature evidencing the effects of regular exercise, including HIIT on blood lipids in obesity [36], only one study, to our knowledge, has demonstrated a hypolipidemic impact of combined exercise with HIIT and Spirulina supplementation in obese male individuals [23]. Fifty-two inactive males with extra body weight participated in the study, and the researchers looked at the individual and combined effects of Spirulina supplementation (4.5 g/day) with or without physical activity (3 days/week) and HIIT (2 days/week). Similar to our findings, they reported increased HDL levels while TC, TG, and LDL levels decreased [23]. This indicates that when a Spirulina supplement is added with HIIT, it can positively impact cardiometabolic factors, which were affected to a lesser degree when supplementation and HIIT were undertaken alone.

The glycemic effects of Spirulina may stem from the presence of fibers, which lowers glucose absorption from the gut [37]. Additionally, phycocyanin, an antioxidant in Spirulina, has enhanced insulin sensitivity through Akt and AMPK signaling [38]. Exercise is well known to facilitate increased insulin sensitivity and glucose regulation through AMPK signaling, leading to an increased expression of GLUT4 and insulin signaling-related proteins [39,40,41]. It is interesting to speculate whether the robust effects on glycemia of combining Spirulina supplementation with HIIT may have been mediated by augmented AMPK activity.

The hypocholesterolemic effect of Spirulina may partly be attributed to its g-linolenic acid (GLA) concentration, which is found in Spirulina as it may reduce hepatic lipid accumulation [37,42,43]. Moreover, phycocyanin likely plays a role in improving lipid profiles in that phycocyanin has been shown to reduce intestinal cholesterol absorption and increase lipoprotein lipase (LPL) that is involved in LDL hydrolysis and subsequently reduce LDL levels [44]. While the underlying mechanisms of lipid-improving effects of exercise are still unclear, increased activation of LPL and reverse cholesterol transport are likely involved, which reduce LDL and increase HDL, respectively. Combined, Spirulina supplementation and HIIT appear to have synergistic effects on glycemic function and blood lipids, producing more significant improvements than when completed alone.

### 4.2. Favorable Modulations of Adipokines

To the authors’ knowledge, this study is the first to investigate the effects of HIIT and Spirulina supplementation on adipokines and cytokines in obesity. We found that Spirulina supplementation and HIIT alone and in combination improve markers of inflammation, with the involvement of HIIT (TSG and TG) producing greater improvements in some markers. For example, we found that circulating CRP was reduced with HIIT and Spirulina supplementation with lower TSG concentrations than TG and SG. We also showed that compared to CG, IL-6, IL-8, and TNF-α were lower in TSG and TG but not SG. For Sema3C and MCP1, HIIT and Spirulina reduced circulating concentrations with no significant differences between SG, TG, and TSG.

Chronic inflammation is a central element in the pathogenesis of systemic cardiometabolic dysfunction that occurs in obesity, including insulin resistance [45,46]. Involved in this process is the secretion of adipokines from adipose tissue and cytokines from immune cells. In non-obese conditions, adipose tissue secretes anti-inflammatory adipokines such as adiponectin; however, in obesity, pro-inflammatory macrophages accumulate, and the adipokine secretory profile transitions to pro-inflammatory [46]. Evidence suggests that this shift in the adipokine secretory profile, in part, results from increases in ROS from mitochondrial dysfunction as well as the infiltration of pro-inflammatory macrophages occurring in obesity [4,47,48]. Therefore, treatments aimed at alleviating oxidative stress and subsequent chronic inflammation represent worthwhile investigation. We showed that 12 weeks of HIIT and Spirulina supplementation improved several markers of adipocytokines and were associated with improved cardiometabolic health markers. While this study did not measure ROS or antioxidant capacity, we hypothesize that improvements in oxidative stress are likely involved in the positive alterations in adipo- and cytokines observed following HIIT and Spirulina supplementation. Though acute exercise increases ROS, regular exercise leads to greater resistance against oxidative damage through increased antioxidant capacity [49]. Similarly, Spirulina exerts antioxidant properties and has been shown to increase total antioxidant status in obese males [35]. Both exercise and Spirulina have also been shown to improve inflammatory status, including when performed together [16,22,35]. Four weeks of Spirulina supplementation of 500 mg/d combined with HIIT increased anti-inflammatory nesfatin-1 and omentin-1 to a greater degree than HIIT alone in overweight and obese women [22].

We hypothesized that the combination of HIIT and Spirulina supplementation would promote improvements in adipocytokines to a greater degree than either alone; however, except for CRP, we found that TSG did not produce greater changes in inflammatory markers compared to TG and SG. This is interesting as TSG produced greater improvements in numerous metabolic and anthropometric markers compared to TG and SG. This indicates that either 12 weeks of HIIT or Spirulina alone is sufficient to lead to positive changes in systemic inflammation; however, the combination of HIIT and Spirulina provides additional clinical benefits. Given this, HIIT and Spirulina supplementation appear to promote positive metabolic health outcomes that may not be mediated through inflammatory signaling, such as ATK-AMPK signaling, as mentioned earlier. Reductions in FAT were similar in TSG, TG, and SG, which may explain the similar alterations in adipocytokines as adiposity status influences inflammatory status in obesity. For example, weight loss reduces pro-inflammatory macrophage infiltration in adipose tissue, reducing systemic inflammation [50].

## 5. Study Limitations

There are various limitations inherent in our investigation. Initially, the processes behind the potential enhancement of adipokine levels by bioactive constituents of Spirolina were not determined. Furthermore, the generalizability of our research is limited due to the exclusion of females in the enrollment of patients. Another limitation of our study is the lack of measurement of blood pressure, heart rate, fat percentage, and MET for the subjects of the present study.

## 6. Conclusions

This study demonstrated that supplementation of the antioxidant Spirulina and HIIT improves anthropometrics, cardiometabolic health markers, and adipocytokine profiles in obese males. Moreover, we showed that while HIIT and Spirulina alone resulted in similar changes in markers of inflammation, the combination of HIIT and Spirulina led to more significant improvements in cardiometabolic health outcomes. This suggests that while HIIT and Spirulina alone can foster improved inflammation in obesity, the combination of both leads to additional beneficial clinical outcomes that appear to be mediated by mechanisms beyond the modulation of obesity-related inflammation.

## Figures and Tables

**Table 1:** Mean (±SD) values of nutritional intake in the four study groups.

	CG		SG		TG		TSG	
	Pre	Post	Pre	Post	Pre	Post	Pre	Post
Energy (kcal/day)	2321 ± 47	2342 ± 56	2354 ± 101	2314 ± 100	2349 ± 117	2297 ± 117	2375 ± 157	2301 ± 126
CHO (g/day)	292 ± 30.4	295 ± 31.3	288.4 ± 25.1	278 ± 26.5	298 ± 41.6	270 ± 37.2	297 ± 39.6	269 ± 30.1
Fat (g/day)	91.2 ± 16.0	92 ± 19.8	95.5 ± 17.7	84 ± 16.2	94.4 ± 19.4	84.1 ± 15.2	91 ± 15.87	75.2 ± 18.3
Protein (g/day)	115 ± 17.0	119 ± 19.3	112 ± 15.5	105 ± 16.6	113 ± 13.8	103 ± 11.7	112 ± 11.5	101 ± 12.5

CG: Control group; SG: Supplement group; TG: Training group; TSG: Training and supplement group.

**Table 2:** Mean (±SD) values of lipid profile, anthropometric, and VO_2peak_ for the four study groups.

	CG		SG		TG		TSG	
	Pre	Post	Pre	Post	Pre	Post	Pre	Post
Body height (cm)	175.7 ± 4.21	-	171.3 ± 4.17	-	173.3 ± 8.16	-	175.2 ± 6.47	-
Body Mass (kg)	101.22 ± 5.27	102.03 ± 2.48	97.81 ± 4.73	97.05 ± 2.45	99.52 ± 10.21	96.22 ± 2.39	101.48 ± 7.95	96.98 ± 1.93 ^a^
BMI (kg/m^2^)	32.77 ± 1.18	33.07 ± 1.40	33.31 ± 0.62	33.13 ± 1.99	33.01 ± 0.76	32.16 ± 2.71	33.00 ± 1.00	31.68 ± 2.18
VO_2peak_ (mL⋅kg^−1^⋅min^−1^)	26.58 ± 1.76	25.71 ± 1.73	26.72 ± 1.36	27.92 ± 2.32 ^a,b^	26.38 ± 1.30	29.93 ± 2.08 ^a,b^	26.46 ± 1.76	30.38 ± 1.97 ^a,b,ab^
HDL (mg/dL)	29.76 ± 6.43	31.67 ± 6.76	30.85 ± 4.68	36.40 ± 5.36 ^a,b^	31.23 ± 4.32	37.23 ± 7.45 ^a,b^	28.19 ± 5.88	42.19 ± 5.48 ^a,b,ab^
LDL (mg/dL)	174.0 ± 13.76	173.5 ± 13.49	172.7 ± 13.91	165.9 ± 11.9 ^a,b^	174.3 ± 10.63	158.2 ± 8.56 ^a,b^	176.4 ± 16.83	151.8 ± 13.87 ^a,b,ab^
TC (mg/dL)	264.2 ± 16.23	269.7 ± 12.24	256.9 ± 20.07	245.7 ± 18.3 ^a,b^	262.3 ± 13.02	245.0 ± 15.0^a,b^	258.9 ± 15.77	240.0 ± 11.14 ^a,b,ab^
TGL (mg/dL)	260.9 ± 15.51	258.9 ± 12.88	262.8 ± 16.75	258.8 ± 13.5 ^a,b^	261.4 ± 20.78	252.0 ± 18.5 ^a,b^	265.8 ± 19.17	253.8 ± 16.48 ^a,b,ab^

CG: control group; SG: supplement group; TG: training group; TSG: training+ supplement group BMI: body mass index; HDL: high-density lipoprotein; LDL: low-density lipoprotein; TC: total cholesterol; TGL: triglyceride. ^a^ Indicates significant differences compared to the pre-values (*p* < 0.05). ^b^ Significant differences compared to the control group (*p* < 0.05). ^ab^ Significant interaction between time and groups (*p* < 0.05).

## Data Availability

The datasets generated for this study are available upon request from the corresponding authors.
